# Surgical counting: design of implementation and maintenance of a standardized evidence-based procedure

**DOI:** 10.1590/0034-7167-2022-0144

**Published:** 2023-02-06

**Authors:** Eduardo Tavares Gomes, Érica Larissa Marinho Souto de Albuquerque, Adélia Cristina Monteiro Pereira, Vilanice Alves de Araujo Püschel

**Affiliations:** I Universidade Federal de Pernambuco, Hospital das Clínicas. Recife, Pernambuco, Brazil.; II Universidade de São Paulo. São Paulo, São Paulo, Brazil.

**Keywords:** Evidence-Based Nursing, Surgicenters, Patient Safety, Implementation Science, Outcome Assessment, Health Care, Enfermería Basada en la Evidencia, Centro Quirúrgicos, Seguridad del Paciente, Ciencia de la Implementación, Evaluación de Resultado en la Atención de Salud, Enfermagem Baseada em Evidências, Centros Cirúrgicos, Segurança do Paciente, Ciência da Implementação, Avaliação de Resultados em Cuidados de Saúde

## Abstract

**Objectives:**

to report the implementation and maintenance of an evidence-based Standard Operating Procedure for surgical counting performed at a teaching hospital.

**Methods:**

a report of a project to implement evidence for surgical counting, carried out at a university hospital in December 2017, and the subsequent cycles for better performance of the implemented organizational document and maintenance of better results until March 2022.

**Results:**

the report is divided into implementation project presentation and four other cycles after implementation, related to maintenance of improvements. It was possible to prepare a Standard Operating Procedure for Surgical Count, train the nursing team, carry out educational intervention for surgical teams.

**Final Considerations:**

there was an improvement in complying with the standardized procedure at the first moment and worsening in the period related to the pandemic. New efforts began again, including a self-instructive online course combined with first-time strategies.

## RESUMO

**Objetivos:**

relatar a implementação e manutenção de um Procedimento Operacional Padronizado baseado em evidências, para contagem cirúrgica, realizado em hospital universitário.

**Métodos:**

trata-se de relato de um projeto de implementação de evidências para contagem cirúrgica, realizado em um hospital universitário, iniciado em dezembro de 2017, e os ciclos subsequentes para melhor desempenho do documento organizacional implementado e manutenção de melhores resultados até março de 2022.

**Resultados:**

o relato está dividido em uma apresentação do projeto de implementação de evidências e mais quatro outros ciclos após a implementação, relacionados à manutenção das melhorias. Foi possível elaborar o documento *Procedimento Operacional Padrão para a Contagem Cirúrgica* no hospital, treinar a equipe de enfermagem, realizar intervenção educativa para as equipes cirúrgicas.

**Considerações Finais:**

houve melhora na adesão ao procedimento padronizado no primeiro momento e piora no período relacionado à pandemia. Novos esforços recomeçaram incluindo curso *online* autoinstrutivo aliado às estratégias do primeiro momento.

## RESUMEN

**Objetivos:**

relatar la implementación y mantenimiento de un Procedimiento Operativo Estandarizado basado en evidencia para el conteo quirúrgico realizado en un hospital universitario.

**Métodos:**

este es un informe de un proyecto de implementación de evidencias para el conteo quirúrgico, realizado en un hospital universitario, iniciado en diciembre de 2017, y los ciclos posteriores para un mejor desempeño del documento organizacional implementado y mantenimiento de mejores resultados hasta marzo de 2022.

**Resultados:**

el informe se divide en una presentación del proyecto de implementación de evidencias y otros cuatro ciclos posteriores a la implementación, relacionados con el mantenimiento de las mejoras. Se logró elaborar el documento Procedimiento Operativo Estándar para el Recuento Quirúrgico en el hospital, capacitar al equipo de enfermería, realizar intervención educativa para los equipos quirúrgicos.

**Consideraciones Finales:**

hubo una mejora en la adherencia al procedimiento estandarizado en el primer momento y un empeoramiento en el período relacionado con la pandemia. Los nuevos esfuerzos comenzaron nuevamente incluyendo un curso en línea autoinstructivo combinado con estrategias para la primera vez.

## INTRODUCTION

The count of items used during surgery is often called surgical counting. It is an essential practice for intraoperative patient safety, considering that, although it is a rare event, retained surgical item (RSI) at the end of surgery is an event that can have serious implications^(1)^.

Counting items, such as needles, compresses and instruments, in the operative field should include the review of fields, hampers and garbage men so that the count of items offered has no discrepancy with the number found at the end^(2)^.

Currently, manual counting is the main one performed in most hospitals, and needles are known to be the least counted items^(3)^. Standardized procedures for surgical counting should be widely disseminated and rigorously complied with between teams, including registration, conference by at least two people (one donned, checking in the operative field, and another off the field, usually the circulator) and at least two moments (when items are included in the field and at the end of surgery)^(4)^. For discrepant counts, before closing the operative wound, after a second count, a radiograph can be performed to identify the item in the cavity^(4)^.

The JBI has published a document that gathers the best evidence for surgical counting. The Evidence Summary Operating Room: Surgical Counts presents recommendations with the respective levels of evidence, based on the principle that there should be high priority for developing more effective and standardized procedures, for preventing the event of forgetfulness/RSI, considering not only the computerization of the process, but human aspects, such as a multidisciplinary approach, construction of standardized institutional procedures and perioperative team training^(1)^.

Although there is evidence available in the literature for best practices, it is still a challenge for surgical centers to ensure the correct counting of surgical items^(1-4)^. Multidisciplinary engagement is a significant challenge, since, for successful counting, the entire surgical team must be involved, and nursing professionals have a leading role in favor of patient safety with the safe execution of the counting process^(4)^. Nursing-led initiatives can not only optimize processes related to surgical counting, but also ensure that personal factors related to surgical professionals or institutional, such as availability of time between surgeries, printouts for records and other resources for counting, interfere minimally with the success of a care instruction for this practice^(4-6)^. An evidence-based guideline should guide the nursing practice of surgical counting, in order to ensure patient and professional safety. The present study brings as guiding question strategies to implement and maintain assets and with good Standard Operating Procedures compliance for evidence-based best practices.

## OBJECTIVES

To report the implementation and maintenance of an evidence-based Standard Operating Procedure for surgical counting performed at a teaching hospital.

## METHODS

This is a report of an evidence implementation project for surgical counting, carried out in a university hospital, initiated in December 2017, and subsequent cycles for better performance of the implemented standardized procedure and maintenance of better results. The university hospital has two operating rooms, a central one with ten operating rooms and an outpatient unit with four rooms, and it serves fifteen surgical specialties, with highly complex procedures.

The report is divided into the evidence implementation project and four other cycles after implementation, related to maintaining improvements.

The implementation project was reported in detail in another publication^(5)^. Considering that, at the time, there were no service statistics on cases and no institutional guidelines for surgical counting, a sample of open surgeries was audited to estimate possible failures in counting and to schedule the first intervention to implement a standardized evidence-based procedure. The following steps were collected from institutional records, mainly from the VigHosp hospital surveillance system.

For the first step, in which the surgeries were audited, and for subsequent data collection, the research was presented to the institution’s Research Ethics Committee, assessed and approved prior to collection.

## RESULTS

### Pre-project organizational situation

The hospital routine did not require the counting of instruments or needles, and surgeons, at the time, could “dismiss” the surgical count. The printed forms used were not suitable for counting recording. Errors and near misses were not recorded as adverse events in the hospital system, and the nursing team was not yet familiar with the system and did not understand the responsibility of the records or their relevance. Discrepant counts were rarely recorded, even in medical records, and radiography was not requested – the team was unaware of this “possibility”.

**Chart 1:** Synthesis of best practice implementation steps, Recife, Pernambuco, Brazil, 2022

**Pre-project step**
Period: before December 2017.
Description: lack of written guidelines and routines, absence of instrumental and sharp counting, failure in compress count. Missing event log.
Results: there are no indicators of the magnitude of the impact of the situation described.
**Evidence implementation project**
Period: December 2017 to March 2018.
Description: baseline audit of 30 surgeries, preparation of the institutional document containing a Standard Operating Procedure (SOP) for surgical counting, educational intervention, final audit of 17 surgeries.
Results: development of the standardized procedure and improved compliance with best practices.
**First cycle: Standardized Operating Procedure prepared and implemented**
Period: April 2018 to March 2020.
Description: increase in records and SOP compliance.
Results: five cases of near miss (counting discrepant) reported.
**Second cycle: peak of the COVID-19 pandemic**
Period: April 2020 to July 2021.
Description: increase in records and decrease in SOP compliance.
Results: three near miss cases (discrepant count) and 4 error records (forgetting surgical items) reported.
**Third cycle: intervention to improve results**
Period: August to November 2021.
Description: self-instructive online course and specific discussions with cases of errors and failures in the SOP steps. Low medical team compliance.
Results: absence of records.
**Fourth cycle: assessment**
Period: December 2021 to March 2022.
Description: monitoring of cases.
Results: absence of records.

### Evidence implementation project

The implementation project had a diagnostic step, followed by an intervention step and an intervention assessment step, with a total duration of six months, between December 2017 and March 2018.

The diagnostic step corresponds to a baseline audit, for which eight audit criteria were developed to assess the surgical counting process steps at the institution. The criteria were taken from a summary of evidence for best practices in surgical counting from the JBI^(1)^. At this step, JBI tools were used, such as the JBI Practical Application of Clinical Evidence System (PACES) and Getting Research into Practice (GRiP), an audit and feedback tool. A team was selected for the project, data collection was carried out from the initial audit of open surgeries and process failures were assessed. The team consisted of four nurses involved in collecting data, carrying out the necessary interventions and assessing the best practices implemented in a new audit cycle with the same criteria used in the first moment. For the audit steps, interviews with technicians and nurses, medical records and participant observation were used. The audit criteria were: 1. The nurse in charge is informed when there is a discrepant count; 2. Fields for incorrect count logging include pertinent event details; 3. A nurse reviews the incorrect count record; 4. There is follow-up of incorrect counting case; 5. A standardized counting approach is strictly performed; 6. A multidisciplinary approach for the surgical team is used for counting; 7. Yields between people in the operating room are limited, to ensure the same team during surgery and on most counts; 8. Reconciliation of surgical count is done before the patient leaves the operating room.

Thirty open surgeries were audited in a period of one month, with several specialties. A matrix was listed containing barriers and strategies to be used to improve the indicators, using the JBI GRiP. The results were discussed with the heads of the surgical specialties, and an evidence-based SOP was developed for surgical counting in the hospital. The SOP was prepared by the researchers, together with nurses from the unit who were involved in the implementation project, using the JBI Evidence Summary as a reference. The SOP underwent adaptations by the elaboration group to the reality of the sector, in order to make it feasible with the available resources^(1)^. When implementing the SOP, banners were made and quick face-to-face meetings were held with all teams, who received pamphlets about the practice. In March 2018, another audit cycle was conducted with the same criteria. In this cycle, the incorporation of technologies that facilitate surgical counting, such as bar code readers, was also attempted, but there was no success due to institutional reasons.

There was low compliance with best practices in five criteria (2, 3, 4, 5, 8), and no surgery had a full-room counting SOP, as it did not include radiography. There was only one case of communication to the student-counting nurse. In 53% (16) surgeries, there was interaction between the surgeon and the technician for the surgical count. The only criterion considered with high strength was 7, with 77% (n=23).

The main barriers to surgical counting identified were lack of knowledge by the team on the relevance of counting, resistance to changing a practice and inadaptation of forms to records. To improve the records, adaptations were made in the form of the Systematization of Perioperative Nursing Care (SAEP - *Sistematização da Assistência de Enfermagem Perioperatória*).

An education program was developed for nurses, nursing technicians, surgeons and residents based on the findings, with banners, pamphlets, individual and group approaches, theoretical classes, with all nursing professionals, to publicize the new surgical counting SOP. The training for nursing professionals was given to 63 participants, lasting 45 minutes. After the education program, a new audit cycle was performed with 17 surgeries. In this cycle, there was greater compliance with instrument and compress count, and six discrepant counts occurred and were recorded, following the SOP. There was an improvement in the counting process, and the SOP was considered implemented and mastered by the nursing team.

### First cycle of Standardized Operating Procedure prepared and implemented

It occurred between April 2018 and March 2020. During this period, there were five cases of discrepant surgical counts with or without reconciliation and no case of forgetting a surgical item, although forgetting a compress can have late consequences and be identified years later. There was no follow-up audit, as of April 2018, for the first criteria considered in the implementation step, considering only that the error and near miss indicators would be enough to warn about SOP compliance. The team defined that a new audit phase could be carried out to investigate the flaws in the SOP that would lead to bad indicators.

There was difficulty in the period in maintaining the training of all those involved, as there is a renewal of residents of surgical specialties every year in March, however, the residents who remained had participated in the campaign and guaranteed SOP compliance in the following year.

### Second cycle of validity of Standardized Operating Procedure

It occurred between April 2020 and July 2021, during the COVID-19 pandemic. During this period, there were sector relocations and closure of the outpatient operating room. Nursing professionals were reassigned to the central operating room to replace the team shortage of recent years. However, the contingent of personnel from the outpatient surgical unit had no experience with major surgeries and no training in surgical counting.

During this period, three cases of discrepant counts with reported reconciliation and four cases of forgetting compresses were identified in the surgical center of which, now in a new location, the obstetric center. The reported cases of reconciliation were in surgeries in which at least one nursing professional participated in the first training to implement the SOP. In cases of forgotten compresses, the circulating professionals had not received training, and the cases were recorded after the complications, with no record in the medical records about the failure to count, as guided by the implemented institutional document. The obstetric center team had not been included in the implementation project.

### Third cycle: intervention to improve results

The monitoring of the cases registered in the previous period motivated a new phase of intervention to disseminate the SOP for surgical count. Between August and November 2021, a self-instructional online course was offered on the hospital’s virtual platform for professionals at the institution. The online format included pre- and post-test, more references and supplementary material (articles on the topic), greater theoretical workload and short videos with simulated case discussions based on cases from the hospital itself. Operating room, obstetric center and medical team professionals were invited to participate. There was low number of medical professionals and nursing professionals at the obstetric center. Moreover, there were brief interventions with the nursing team to discuss the cases found with each new notification. The online course was made available on the platform, being included in the admission training for the unit, and a version was included in the institution’s patient safety course, mandatory for residents and new professionals.

### Fourth cycle: assessment

From the beginning of the previous cycle, in August 2021 until the month of March 2022, there was no record of forgetting compresses or discrepant counts – near misses.

## DISCUSSION

Current evidence of higher recommendation to avoid RSI events involves more light, behavioral technologies than harsh technologies^(1)^. Regardless of manual or computerized instrument count, team training, considering the multidisciplinary surgical team, is decisive for the event avoidability^(1-2)^. Furthermore, the dissemination of a culture of surveillance and notification is decisive for monitoring errors and near misses and for directing interventions^(2)^.

This project aimed to implement in the service best practices of surgical counting to promote safety for professionals and patients. It was observed that, although the initial efforts and their positive results, the maintenance of efforts is of paramount importance. Personnel renewal should be considered and, in the case of a university hospital, this is a very relevant factor due to the participation of students and residents in the surgery, renewing the team periodically. Although the JBI methodology recommends more periodic follow-up audits using the same baseline audit criteria, the main indicators of the need to reinforce SOP compliance in the present study were the error and near miss records. The outcomes towards which efforts are directed are key indicators of the need for strategies to maintain implementation results; however, the application of the same audit criteria allows more accuracy to identify which points in the process are more flawed and need more attention.

One of the positive results was the SOP preparation for hospital surgical counting. Even with decades of existence, until the beginning of this project, the hospital did not have a SOP like this. The relevance of SOP lies not only in improving safety, but in influencing the training of medical and nursing students and residents, who are potential disseminators of evidence-based practice learned beyond the hospital walls.

Another positive result was the reformulation of the SAEP registration form. The current instrument is the third version and is suitable for recording all events from needle, compress and instrument counts to discrepant counts.

Evidence for surgical counting is currently available and there are many discussions and efforts towards implementing best practices^(1, 2, 3, 4, 5, 6)^. To this end, institutional documents must be prepared in order to standardize the time to carry out the counts, the form of registration, actions to be taken in discrepant count and the relevant flows involved^(6)^.

Another initiative with a strategy similar to the present implementation project achieved a 50% reduction in the number of incorrect and discrepant counts^(6)^. In the study presented here, it is not possible to directly state the percentage of reduction of near misses found, since the practice of notification was encouraged, which was not carried out before. However, the research team, over the course of that year, was able to verify that the educational intervention improved the outcomes of compliance with the protocol, improved count records, improved the investigation of discrepant counts and the use of intraoperative radiography in the investigation.

As a positive point, we highlight the change in culture that counting is a waste of time and the empowerment of nursing technicians to stop failures in the process. Emergency surgeries have the highest failure rate, however the study’s surgical center only attends to elective surgeries, and the hospital does not have an emergency service^(7)^.

There are still barriers in the service to requesting intraoperative radiography, with some professionals trying to avoid wasting time for the next surgery. However, it is clear to all nursing that it is not an option not to follow the SOP guidelines. Some professionals still try to dispense with counting instruments, claiming it is not an important step, and counting sharps has lower compliance. There is an incentive for the presence of a nurse in the room at the end of the surgery, since it is known that the presence of nurses increases SOP compliance and reduces noise and distractions^(3, 7-8)^.

### Study limitations

As limitations of this study, it is considered not including radiology professionals in the training steps, although there are international recommendations for them to be included^(6)^. During the follow-up period (December 2017 to March 2022), the hospital was unable to include technologies to improve the surgical counting process, such as the use of bar codes, scanners, radiofrequency detectors, although they are new technologies in several countries, and there is already evidence for their use, or even electronic medical records for recording intraoperative activities^(1, 6, 9)^. Although errors and near misses have been reported, it is not possible to take the number of notifications as an absolute value for the number of cases in the outliers, considering that item recount takes place several times a week with reconciliation and few cases are reported. The reported cases of discrepant counts were only those where there was no reconciliation or where there were difficulties in counting with the surgical team. Finally, the feedback of the results to the teams can be cited as a limitation of this study, since the return of notified cases is made only to the head of each specialty involved, and there is no wide dissemination of cases, case studies, discussions and dissemination of statistical data. The discussion of cases is of paramount importance to complete the educational process^(6, 10)^.

### Contributions to nursing

With the project of implementation and maintenance of a standardized procedure based on evidence, it was possible to collaborate with the SOP elaboration for surgical counting in the hospital, adapt the forms, train the nursing team, carry out educational interventions for the surgical teams, foster the culture of surgical counting for patient safety and empower nursing technicians to take a leading role in the process. Additionally, the dissemination of this work, the result of years of collective efforts, can contribute to motivating improvement processes in other surgical centers and expanding the safety culture among operating room professionals.

## FINAL CONSIDERATIONS

Surgical counting should be implemented as a best practice in an evidence-based operating room and international guidelines. The initial phase of implementing evidence-based best practices struggled at a time when there was no routine, no written guidelines, and no culture focused on the relevance of surgical counting and event recording. The JBI methodology for implementing best practices based on evidence was instrumental in initiating and guiding this reported process.

With the implementation project and the SOP assessment and maintenance cycles, there was a change in the service’s culture, with broad awareness and promotion of critical thinking, in addition to mere instrumentation.

There was an improvement in SOP compliance at the first moment and worsening in the period related to the pandemic. New efforts have resumed, including self-instructing online course coupled with first-time strategies.

## References

[1] McArthur A (2016). Operating room: surgical counts surname. Evidence Summary.

[2] Nelson P (2021). Incorrect surgical counts: a potential for retained surgical items. J Dr Nurs Pract.

[3] Freitas PS, Mendes KDS, Galvão CM (2016). Surgical count process: evidence for patient safety. Rev Gaúcha Enferm.

[4] Fang J, Yuan X, Fan L, Du M, Sui W, Ma W (2021). Risk factors for incorrect surgical count during surgery: an observational study. Int J Nurs Pract.

[5] Gomes ET, Galvão MCB, Shimoda GT, Oliveira LB, Araújo Püschel VA (2021). Surgical counts in open abdominal and pelvic surgeries in a university hospital: a best practice implementation project. JBI Evid Implement.

[6] Norton E, Martin C, Micheli A (2012). Patients count on it: An initiative to reduce incorrect counts and prevent retained surgical items. AORN J.

[7] Moffatt-Bruce SD, Cook CH, Steinberg SM, Stawicki SP (2014). Risk factors for retained surgical items: a meta-analysis and proposed risk stratification system. J Surg Res.

[8] Bubric KA, Biesbroek SL, Laberge JC, Martel JA, Litvinchuk SD (2021). Prevalence and Characteristics of Interruptions and Distractions During Surgical Counts. Jt Comm J Qual Patient Saf.

[9] Frasier LL, Pavuluri Quamme SR, Wiegmann D, Greenberg CC (2020). Evaluation of intraoperative hand-off frequency, duration, and context: a mixed methods analysis. J Surg Res.

[10] Hibbert PD, Thomas MJW, Deakin A, Runciman WB, Carson-Stevens A, Braithwaite J (2020). A qualitative content analysis of retained surgical items: learning from root cause analysis investigations. Int J Qual Health Care.

